# Supplementary material for: Modeling glioblastoma heterogeneity as a dynamic network of cell states
Source: Mol Syst Biol. 2021 Sep 16;17(9):e10105. doi: 10.15252/msb.202010105 (PMC8444284; doi:10.15252/msb.202010105)
Supplement: Supplementary file 6 — Source Data for Figure 5 [file MSB-17-e10105-s004.zip › Figure5A_sourcedata/GSEA_3017/nosigdb_stateF.GseaPreranked.1622795015644/RICHARDS_NOWAKOWSKI_SCIENCE_2017_MICROGLIA_UPREG.html]

Details for gene set RICHARDS\_NOWAKOWSKI\_SCIENCE\_2017\_MICROGLIA\_UPREG[GSEA]

|  || Dataset | state33017 |
| Phenotype | NoPhenotypeAvailable |
| Upregulated in class | na\_pos |
| GeneSet | RICHARDS\_NOWAKOWSKI\_SCIENCE\_2017\_MICROGLIA\_UPREG |
| Enrichment Score (ES) | 0.38943836 |
| Normalized Enrichment Score (NES) | 2.8182552 |
| Nominal p-value | 0.0 |
| FDR q-value | 0.0 |
| FWER p-Value | 0.0 |
Table: GSEA Results Summary

  

Fig 1: Enrichment plot: RICHARDS\_NOWAKOWSKI\_SCIENCE\_2017\_MICROGLIA\_UPREG      
 Profile of the Running ES Score & Positions of GeneSet Members on the Rank Ordered List

  

| PROBE | GENE SYMBOL | GENE\_TITLE | RANK IN GENE LIST | RANK METRIC SCORE | RUNNING ES | CORE ENRICHMENT || 1 | NEAT1 |  |  | 0 | 2.864 | 0.0268 | Yes |
| 2 | CDKN1A |  |  | 4 | 2.089 | 0.0449 | Yes |
| 3 | CCL2 |  |  | 7 | 1.774 | 0.0606 | Yes |
| 4 | CST3 |  |  | 15 | 1.273 | 0.0691 | Yes |
| 5 | APOE |  |  | 17 | 1.244 | 0.0803 | Yes |
| 6 | FTH1 |  |  | 19 | 1.203 | 0.0910 | Yes |
| 7 | PNRC1 |  |  | 23 | 1.070 | 0.0996 | Yes |
| 8 | SLC1A3 |  |  | 26 | 1.058 | 0.1085 | Yes |
| 9 | SDCBP |  |  | 27 | 1.054 | 0.1184 | Yes |
| 10 | FTL |  |  | 32 | 1.036 | 0.1262 | Yes |
| 11 | ITPR2 |  |  | 34 | 1.024 | 0.1353 | Yes |
| 12 | TNFRSF21 |  |  | 36 | 1.016 | 0.1443 | Yes |
| 13 | A2M |  |  | 42 | 0.974 | 0.1510 | Yes |
| 14 | SAT1 |  |  | 45 | 0.957 | 0.1589 | Yes |
| 15 | CPEB4 |  |  | 46 | 0.953 | 0.1679 | Yes |
| 16 | ABCA1 |  |  | 48 | 0.938 | 0.1762 | Yes |
| 17 | BAX |  |  | 50 | 0.933 | 0.1844 | Yes |
| 18 | SQSTM1 |  |  | 54 | 0.914 | 0.1915 | Yes |
| 19 | SPP1 |  |  | 57 | 0.898 | 0.1989 | Yes |
| 20 | LPL |  |  | 59 | 0.874 | 0.2066 | Yes |
| 21 | GLUL |  |  | 69 | 0.837 | 0.2101 | Yes |
| 22 | SERPINB6 |  |  | 83 | 0.791 | 0.2111 | Yes |
| 23 | UBL3 |  |  | 88 | 0.778 | 0.2164 | Yes |
| 24 | MALAT1 |  |  | 90 | 0.773 | 0.2232 | Yes |
| 25 | IFITM2 |  |  | 103 | 0.749 | 0.2243 | Yes |
| 26 | B2M |  |  | 110 | 0.736 | 0.2283 | Yes |
| 27 | NPC2 |  |  | 114 | 0.728 | 0.2336 | Yes |
| 28 | GNG7 |  |  | 115 | 0.726 | 0.2404 | Yes |
| 29 | BRI3 |  |  | 121 | 0.716 | 0.2447 | Yes |
| 30 | BTG2 |  |  | 124 | 0.708 | 0.2504 | Yes |
| 31 | FCGR2A |  |  | 134 | 0.688 | 0.2524 | Yes |
| 32 | MAF |  |  | 136 | 0.688 | 0.2584 | Yes |
| 33 | HMOX1 |  |  | 142 | 0.684 | 0.2623 | Yes |
| 34 | ITM2C |  |  | 160 | 0.649 | 0.2601 | Yes |
| 35 | ATP1B1 |  |  | 167 | 0.635 | 0.2631 | Yes |
| 36 | NAMPT |  |  | 168 | 0.634 | 0.2690 | Yes |
| 37 | ATP2B1 |  |  | 170 | 0.632 | 0.2744 | Yes |
| 38 | RSRP1 |  |  | 173 | 0.623 | 0.2793 | Yes |
| 39 | MTUS1 |  |  | 178 | 0.614 | 0.2831 | Yes |
| 40 | ITM2B |  |  | 181 | 0.610 | 0.2878 | Yes |
| 41 | NINJ1 |  |  | 182 | 0.610 | 0.2935 | Yes |
| 42 | ATP1A1 |  |  | 185 | 0.605 | 0.2982 | Yes |
| 43 | MTRNR2L8 |  |  | 186 | 0.605 | 0.3039 | Yes |
| 44 | CEBPB |  |  | 190 | 0.601 | 0.3080 | Yes |
| 45 | HERPUD1 |  |  | 197 | 0.590 | 0.3106 | Yes |
| 46 | ATP2A2 |  |  | 210 | 0.582 | 0.3102 | Yes |
| 47 | APOBEC3C |  |  | 224 | 0.572 | 0.3092 | Yes |
| 48 | SLC2A3 |  |  | 228 | 0.569 | 0.3130 | Yes |
| 49 | HTRA1 |  |  | 229 | 0.568 | 0.3184 | Yes |
| 50 | CEBPD |  |  | 234 | 0.566 | 0.3217 | Yes |
| 51 | SRGAP2C |  |  | 238 | 0.563 | 0.3255 | Yes |
| 52 | NLRP1 |  |  | 257 | 0.547 | 0.3218 | Yes |
| 53 | ARL6IP5 |  |  | 264 | 0.542 | 0.3240 | Yes |
| 54 | GPR137B |  |  | 265 | 0.541 | 0.3290 | Yes |
| 55 | TFRC |  |  | 270 | 0.537 | 0.3321 | Yes |
| 56 | PLA2G16 |  |  | 284 | 0.523 | 0.3306 | Yes |
| 57 | TPP1 |  |  | 286 | 0.521 | 0.3350 | Yes |
| 58 | ASAH1 |  |  | 293 | 0.515 | 0.3369 | Yes |
| 59 | ATP6V0E1 |  |  | 303 | 0.510 | 0.3373 | Yes |
| 60 | LAMP2 |  |  | 308 | 0.506 | 0.3401 | Yes |
| 61 | NRP2 |  |  | 313 | 0.503 | 0.3428 | Yes |
| 62 | IL6ST |  |  | 321 | 0.500 | 0.3441 | Yes |
| 63 | RNF13 |  |  | 330 | 0.496 | 0.3448 | Yes |
| 64 | CSF1 |  |  | 333 | 0.495 | 0.3484 | Yes |
| 65 | PLD3 |  |  | 337 | 0.494 | 0.3516 | Yes |
| 66 | ITGAV |  |  | 344 | 0.490 | 0.3533 | Yes |
| 67 | MMP2 |  |  | 357 | 0.485 | 0.3519 | Yes |
| 68 | LIPA |  |  | 364 | 0.483 | 0.3535 | Yes |
| 69 | ID2 |  |  | 366 | 0.483 | 0.3575 | Yes |
| 70 | HSPA5 |  |  | 373 | 0.480 | 0.3591 | Yes |
| 71 | GATM |  |  | 375 | 0.480 | 0.3631 | Yes |
| 72 | CTSL |  |  | 376 | 0.479 | 0.3676 | Yes |
| 73 | PLXNB2 |  |  | 398 | 0.471 | 0.3617 | Yes |
| 74 | SORL1 |  |  | 402 | 0.469 | 0.3646 | Yes |
| 75 | METTL7A |  |  | 403 | 0.469 | 0.3690 | Yes |
| 76 | SLC3A2 |  |  | 411 | 0.466 | 0.3700 | Yes |
| 77 | PSAP |  |  | 418 | 0.463 | 0.3713 | Yes |
| 78 | DNASE2 |  |  | 420 | 0.462 | 0.3752 | Yes |
| 79 | LAMP1 |  |  | 440 | 0.455 | 0.3701 | Yes |
| 80 | MAP1LC3B |  |  | 445 | 0.453 | 0.3724 | Yes |
| 81 | DDIT3 |  |  | 447 | 0.451 | 0.3762 | Yes |
| 82 | ATP6AP1 |  |  | 450 | 0.450 | 0.3794 | Yes |
| 83 | GAS6 |  |  | 463 | 0.445 | 0.3777 | Yes |
| 84 | SEC14L1 |  |  | 465 | 0.445 | 0.3814 | Yes |
| 85 | TIPARP |  |  | 472 | 0.444 | 0.3826 | Yes |
| 86 | GRN |  |  | 474 | 0.443 | 0.3862 | Yes |
| 87 | JUND |  |  | 478 | 0.442 | 0.3889 | Yes |
| 88 | LPIN2 |  |  | 488 | 0.438 | 0.3886 | Yes |
| 89 | YBX3 |  |  | 509 | 0.431 | 0.3829 | Yes |
| 90 | ATP6V0D1 |  |  | 512 | 0.430 | 0.3859 | Yes |
| 91 | ATP6AP2 |  |  | 514 | 0.430 | 0.3894 | Yes |
| 92 | PARP14 |  |  | 541 | 0.417 | 0.3806 | No |
| 93 | CFLAR |  |  | 569 | 0.408 | 0.3712 | No |
| 94 | RAB31 |  |  | 580 | 0.404 | 0.3701 | No |
| 95 | TMSB4X |  |  | 590 | 0.401 | 0.3694 | No |
| 96 | FOS |  |  | 592 | 0.401 | 0.3727 | No |
| 97 | IRS2 |  |  | 595 | 0.400 | 0.3755 | No |
| 98 | IER5 |  |  | 605 | 0.396 | 0.3748 | No |
| 99 | GABARAPL1 |  |  | 654 | 0.384 | 0.3549 | No |
| 100 | NFE2L2 |  |  | 663 | 0.381 | 0.3545 | No |
| 101 | DOCK4 |  |  | 668 | 0.380 | 0.3561 | No |
| 102 | ATP6V1F |  |  | 685 | 0.374 | 0.3518 | No |
| 103 | TNFRSF1A |  |  | 686 | 0.374 | 0.3553 | No |
| 104 | DNAJC3 |  |  | 689 | 0.374 | 0.3578 | No |
| 105 | RGS16 |  |  | 691 | 0.373 | 0.3608 | No |
| 106 | S100A11 |  |  | 692 | 0.373 | 0.3643 | No |
| 107 | MEF2C |  |  | 696 | 0.372 | 0.3663 | No |
| 108 | CSTB |  |  | 714 | 0.368 | 0.3614 | No |
| 109 | ADAM9 |  |  | 722 | 0.365 | 0.3614 | No |
| 110 | GAL3ST4 |  |  | 738 | 0.362 | 0.3575 | No |
| 111 | COQ10B |  |  | 749 | 0.359 | 0.3559 | No |
| 112 | RBMS1 |  |  | 761 | 0.355 | 0.3539 | No |
| 113 | SIPA1L2 |  |  | 766 | 0.354 | 0.3552 | No |
| 114 | FLCN |  |  | 772 | 0.353 | 0.3561 | No |
| 115 | SLC38A2 |  |  | 785 | 0.350 | 0.3535 | No |
| 116 | TPT1 |  |  | 786 | 0.350 | 0.3568 | No |
| 117 | UBC |  |  | 792 | 0.348 | 0.3576 | No |
| 118 | KCTD12 |  |  | 808 | 0.345 | 0.3535 | No |
| 119 | FCGRT |  |  | 814 | 0.344 | 0.3542 | No |
| 120 | RHOQ |  |  | 843 | 0.339 | 0.3437 | No |
| 121 | ZFP36L2 |  |  | 850 | 0.336 | 0.3439 | No |
| 122 | STAT3 |  |  | 880 | 0.330 | 0.3328 | No |
| 123 | WSB1 |  |  | 882 | 0.329 | 0.3354 | No |
| 124 | ARL4A |  |  | 889 | 0.328 | 0.3355 | No |
| 125 | GSTM4 |  |  | 909 | 0.324 | 0.3292 | No |
| 126 | PEAK1 |  |  | 918 | 0.323 | 0.3283 | No |
| 127 | BHLHE40 |  |  | 928 | 0.321 | 0.3269 | No |
| 128 | TLN2 |  |  | 944 | 0.318 | 0.3226 | No |
| 129 | ST3GAL6 |  |  | 946 | 0.318 | 0.3251 | No |
| 130 | CD81 |  |  | 947 | 0.318 | 0.3280 | No |
| 131 | USP2 |  |  | 953 | 0.317 | 0.3286 | No |
| 132 | SCPEP1 |  |  | 971 | 0.314 | 0.3232 | No |
| 133 | HIF1A |  |  | 972 | 0.314 | 0.3261 | No |
| 134 | FMN1 |  |  | 1002 | 0.310 | 0.3148 | No |
| 135 | SRGAP2B |  |  | 1006 | 0.309 | 0.3162 | No |
| 136 | CPVL |  |  | 1019 | 0.307 | 0.3132 | No |
| 137 | TCEAL4 |  |  | 1052 | 0.299 | 0.3004 | No |
| 138 | ARHGEF6 |  |  | 1054 | 0.299 | 0.3027 | No |
| 139 | PELI1 |  |  | 1059 | 0.298 | 0.3035 | No |
| 140 | INPP5D |  |  | 1069 | 0.297 | 0.3019 | No |
| 141 | ATP6V0A1 |  |  | 1082 | 0.295 | 0.2988 | No |
| 142 | CYLD |  |  | 1085 | 0.295 | 0.3005 | No |
| 143 | SPECC1 |  |  | 1089 | 0.294 | 0.3018 | No |
| 144 | RNF213 |  |  | 1091 | 0.294 | 0.3041 | No |
| 145 | SH3BP2 |  |  | 1099 | 0.293 | 0.3034 | No |
| 146 | SKAP2 |  |  | 1103 | 0.292 | 0.3047 | No |
| 147 | LGMN |  |  | 1110 | 0.291 | 0.3045 | No |
| 148 | PLEKHA2 |  |  | 1113 | 0.290 | 0.3062 | No |
| 149 | PIK3R1 |  |  | 1121 | 0.289 | 0.3055 | No |
| 150 | KDM6B |  |  | 1139 | 0.286 | 0.2998 | No |
| 151 | MFSD1 |  |  | 1144 | 0.286 | 0.3006 | No |
| 152 | RNF19B |  |  | 1148 | 0.285 | 0.3018 | No |
| 153 | RAB1A |  |  | 1201 | 0.279 | 0.2789 | No |
| 154 | ADAM17 |  |  | 1215 | 0.277 | 0.2751 | No |
| 155 | ATP6V1B2 |  |  | 1219 | 0.277 | 0.2762 | No |
| 156 | ANKRD10 |  |  | 1231 | 0.276 | 0.2734 | No |
| 157 | PTPRE |  |  | 1243 | 0.275 | 0.2706 | No |
| 158 | CTSD |  |  | 1245 | 0.275 | 0.2727 | No |
| 159 | NCKAP5 |  |  | 1279 | 0.271 | 0.2591 | No |
| 160 | SGK1 |  |  | 1283 | 0.270 | 0.2601 | No |
| 161 | IFNGR1 |  |  | 1289 | 0.269 | 0.2602 | No |
| 162 | SDCCAG8 |  |  | 1291 | 0.269 | 0.2622 | No |
| 163 | NEU1 |  |  | 1296 | 0.269 | 0.2628 | No |
| 164 | ARID3A |  |  | 1332 | 0.264 | 0.2481 | No |
| 165 | ABHD3 |  |  | 1382 | 0.259 | 0.2265 | No |
| 166 | MALT1 |  |  | 1387 | 0.258 | 0.2270 | No |
| 167 | EEPD1 |  |  | 1397 | 0.257 | 0.2250 | No |
| 168 | PDE4B |  |  | 1404 | 0.256 | 0.2245 | No |
| 169 | AFF1 |  |  | 1406 | 0.256 | 0.2264 | No |
| 170 | CITED2 |  |  | 1433 | 0.254 | 0.2160 | No |
| 171 | MGAT4A |  |  | 1448 | 0.252 | 0.2115 | No |
| 172 | JUNB |  |  | 1466 | 0.251 | 0.2055 | No |
| 173 | APLP2 |  |  | 1468 | 0.250 | 0.2074 | No |
| 174 | HSPH1 |  |  | 1495 | -0.258 | 0.1971 | No |
| 175 | CBFB |  |  | 1499 | -0.259 | 0.1980 | No |
| 176 | GSTO1 |  |  | 1525 | -0.265 | 0.1883 | No |
| 177 | HSP90AA1 |  |  | 1535 | -0.268 | 0.1864 | No |
| 178 | PFN1 |  |  | 1582 | -0.282 | 0.1665 | No |
| 179 | HBEGF |  |  | 1583 | -0.282 | 0.1691 | No |
| 180 | ZFP36L1 |  |  | 1590 | -0.284 | 0.1688 | No |
| 181 | EHD4 |  |  | 1620 | -0.290 | 0.1573 | No |
| 182 | UBE2D3 |  |  | 1669 | -0.306 | 0.1367 | No |
| 183 | TACC1 |  |  | 1701 | -0.319 | 0.1245 | No |
| 184 | MRPL14 |  |  | 1714 | -0.323 | 0.1216 | No |
| 185 | PABPC4 |  |  | 1728 | -0.329 | 0.1183 | No |
| 186 | HSPD1 |  |  | 1742 | -0.336 | 0.1151 | No |
| 187 | TPM3 |  |  | 1780 | -0.353 | 0.1003 | No |
| 188 | TMEM107 |  |  | 1789 | -0.357 | 0.0997 | No |
| 189 | LGALS1 |  |  | 1799 | -0.362 | 0.0987 | No |
| 190 | LIMS1 |  |  | 1807 | -0.365 | 0.0987 | No |
| 191 | RPL35 |  |  | 1820 | -0.375 | 0.0963 | No |
| 192 | C12orf75 |  |  | 1832 | -0.380 | 0.0945 | No |
| 193 | CREM |  |  | 1842 | -0.385 | 0.0937 | No |
| 194 | PLEKHO1 |  |  | 1858 | -0.393 | 0.0900 | No |
| 195 | HIST1H3D |  |  | 1867 | -0.398 | 0.0898 | No |
| 196 | TCP1 |  |  | 1875 | -0.401 | 0.0901 | No |
| 197 | FKBP4 |  |  | 1903 | -0.426 | 0.0809 | No |
| 198 | HMGA1 |  |  | 1915 | -0.433 | 0.0796 | No |
| 199 | EIF5 |  |  | 1923 | -0.439 | 0.0803 | No |
| 200 | CLIC1 |  |  | 1949 | -0.454 | 0.0723 | No |
| 201 | DDX39A |  |  | 1957 | -0.458 | 0.0731 | No |
| 202 | HSP90AB1 |  |  | 1963 | -0.461 | 0.0750 | No |
| 203 | CDV3 |  |  | 1994 | -0.487 | 0.0649 | No |
| 204 | TRA2B |  |  | 1998 | -0.490 | 0.0680 | No |
| 205 | HNRNPUL1 |  |  | 2009 | -0.501 | 0.0678 | No |
| 206 | RANGAP1 |  |  | 2011 | -0.502 | 0.0720 | No |
| 207 | IER2 |  |  | 2034 | -0.524 | 0.0661 | No |
| 208 | EIF5A |  |  | 2043 | -0.529 | 0.0672 | No |
| 209 | EPB41L2 |  |  | 2067 | -0.576 | 0.0613 | No |
| 210 | DDX21 |  |  | 2107 | -0.659 | 0.0484 | No |
| 211 | CKS2 |  |  | 2115 | -0.694 | 0.0515 | No |
| 212 | ODC1 |  |  | 2128 | -0.718 | 0.0523 | No |
| 213 | MIS18BP1 |  |  | 2183 | -0.903 | 0.0343 | No |
Table: GSEA details [plain text format]

  

Fig 2: RICHARDS\_NOWAKOWSKI\_SCIENCE\_2017\_MICROGLIA\_UPREG: Random ES distribution      
 Gene set null distribution of ES for **RICHARDS\_NOWAKOWSKI\_SCIENCE\_2017\_MICROGLIA\_UPREG**
